# The Reckless Sacrifice of Tooth Substance in Devitalized Teeth

**Published:** 1885-08

**Authors:** W. N. Morrison


ARTICLE IV.

### THE RECKLESS SACRIFICE OF TOOTH SUBSTANCE IN DEVITALIZED TEETH.

BY W. N. MORRISON, D.D.S.

[Read before the Southern Dental Association, New Orleans, April, 1885.]

There is an opereration of great importance that I have never seen discussed before in our societies, or alluded to in the professional literature, that I would like to call attention to. That is the reckless sacrifice of tooth substance in devitalized teeth. By some of the old operators we have been instructed when opening dead teeth to cut freely, and to make the opening large, so we can see into every part of the cavity, and in many instances drill out the individual root canals, removing solid dentine from the best part of the crown of the tooth, unnecessarily weakening the tooth in the place where it should have the most strength, burring it out to the extent of one-eighth of an inch or more in diameter. In many instances these roots are filled with cotton, and this cavity is filled full of whatever material is used, leaving only a thin, frail shell of dentine between the filling and the enamel. Soon follows the breaking down of these walls, by the ordinary use of the jaw in mastication. I have always felt that there was no excuse for this wholesale destruction of so important a part of the teeth, and have been working in a line to save all the crown substance of dead teeth possible and with the method of root filling which I have used for many years, I find the range of possibilities in this direction very great. Of all materials used or recommended for canal fillings, I have never found anything equal to pure gold wire, of size to correspond to the size of the different canals. The method in which I apply it is described in detail in the *Missouri Dental Journal* for 1874, page 51.

By following out the method described in this article, it will be possible for almost any ordinarily skillful operator to fill the canals of the molars through an opening not to exceed one-sixteenth of an inch in diameter. This opening must be made directly in the center of the crown, with a drill of that size, and then, with a round, or truncated-cone-bur, the sharp angle of the pulp-chamber at the lower end of this hole is slightly funneled, by which access is had with small steel broaches, for the removal of the dead pulp, and the cleansing and treatment, if necessary, of these canals with medicated cotton. And just here I wish to give a word of warning against the over-treatment of canals where the pulp tissue is entirely removed. When I am certain that the root vessels are all removed, and there is no inflammation in the peridental membrane, I fill such canals immediately, often leaving one or two of the other canals for subsequent treatment, where I am not certain the tissue has been removed.

I recently had a case of a lower first molar, with a very large mesial cavity of black decay, which progressed slowly, and was of long standing; a gentleman about forty-five years of age. Being uncertain as to whether the pulp was in a healthy condition enough to live, I gave it the benefit of the doubt, and put in a large filling. The tooth remained comfortable for several weeks, and finally it began to have neuralgic disturbances on that side, with flashes of pain occasionally, and extreme sensitiveness to thermal changes, both of the air and food, the pain recurring nearly always at night. The gentleman exercised a great deal of patience with it, and was equally anxious with me to keep the pulp alive; but one morning he came to me and said he could endure it no longer. The tooth was quite sore, and I found enough peridental inflammation to warrant me to make an opening into the pulp, which I did through the top of the crown, not removing the good mesial filling, but made a small hole, not exceeding one-sixteenth of an inch. I removed the vessels from one of the roots without much pain. Those of the other root were exceedingly sensitive, and I devitalized them with arsenious acid. In removing the pulp, I discovered quite a nodule of secondary dentine, about the size of quail shot, in the pulp chamber. You will readily appreciate my dilemna. With a small, sharp, barbed broach, I tore all of the pulp tissue from this nodule that would catch on the broach, and pulled it toward the orifice, and as the broach would draw it through, it would disengage itself from this little nodule, which I could feel was perfectly smooth. After I had the vessels all removed from these roots, I rolled the nodule forward over the orifice in the mesial root, and with my gold wire nicely fitted to the distal root and with a little chloro-percha to fill the interstices between the gold wire and the root walls, I filled the distal root solid and secure to its end. Then with the instrument I rolled this nodule back over the orifice and filled the mesial root in the same manner.

In all my operations, I endeavor to accommodate the instrument to the case, instead of cutting valuable tooth substance to suit bungling instruments. In lateral incisors, for instance, where a dead pulp is diagnosed, I drill an opening exactly on a line with the pulp canal that will just admit the size of wire that I use for such canals, and no larger.

